# Peer-to-peer: The Social Transmission of Symptoms Online

**DOI:** 10.1093/abm/kaac081

**Published:** 2023-04-10

**Authors:** Winston Tan, Brydee Pickup, Kate Faasse, Ben Colagiuri, Kirsten Barnes

**Affiliations:** School of Psychology, University of Sydney, Camperdown, NSW, Australia; School of Psychology, University of Sydney, Camperdown, NSW, Australia; School of Psychology, University of New South Wales, Kensington, NSW, Australia; School of Psychology, University of Sydney, Camperdown, NSW, Australia; School of Psychology, University of Sydney, Camperdown, NSW, Australia

**Keywords:** Social learning, Health, Social influence, Virtual reality, Social transmission

## Abstract

**Background:**

Social learning can be highly adaptive—for example, avoiding a hotplate your friend just burnt themselves on—but it has also been implicated in symptom transmission. Social learning is particularly pertinent given the rapid increase in the use of online mediums for social interaction. Yet, little is known about the social transmission of symptoms online or social chains extending further than a single model–observer interaction.

**Purpose:**

To explore whether socially induced symptoms could be propagated through a three-generation social transmission chain in an online setting.

**Methods:**

We explored the social transmission of cybersickness following a virtual reality (VR) experience through online webcam interactions. One hundred and seventy-seven adults viewed a VR video in one of four links along a social transmission chain, after: viewing an actor model cybersickness to the VR video (First-Generation); viewing the First-Generation participant undergo VR (Second-Generation); viewing the Second-Generation participant undergo VR (Third-Generation); or naïve (Control).

**Results:**

Cybersickness was strongest in First-Generation participants, indicating social transmission from the model. This was mediated by expectancy and anxiety. Whether or not subsequent generations experienced cybersickness depended on what the observed participant verbally reported, which is consistent with social transmission.

**Conclusions:**

Results demonstrate that symptoms can be readily transmitted online, and that expectancy and anxiety are involved. Although it is inconclusive as to whether symptoms can propagate along a social transmission chain, there is some evidence of protection from symptoms when a model who does not report any symptoms is observed. As such, this research highlights the role of social transmission in the modulation of symptoms through virtual mediums.

## Teaser

Individuals who witnessed another person experiencing side effects from virtual reality reported more severe side effects following their own subsequent virtual reality experience relative to individuals who did not observe another person

## Peer-to-Peer: The Social Transmission of Symptoms Online

Mobile apps monitor our health [1], online communication facilitates work [2], and virtual reality (VR) has been adopted in medical, education, and commercial settings [3, 4]. Yet, despite the increasing amount of time spent interacting online, a paucity of research exists concerning whether virtual observation of other people’s experiences (“social observation”) can negatively impact how we feel ourselves, particularly in terms of symptoms. Nascent research (see Ref. [5] for review) suggests that online social transmission of symptoms may be possible, but no studies have examined this.

Emerging research proposes that social learning can induce negative expectations that exacerbate symptoms [6]. Here, social learning refers to an individual’s own experiences and/or behaviors being influenced by the observation of another individual’s experiences and/or behaviors [7]. Modulation of symptoms via the assimilation of social information has been demonstrated in pain (e.g., [8–11], headache [12], and nausea [13]). Concerningly, this social influence is far-reaching, with socially learned symptoms observed across a range of scenarios, including for medication [14, 15], painful procedures [11], and environmental toxins [13]. Social learning has also been implicated at the community level (e.g., “wind turbine syndrome” and “electromagnetic hypersensitivity”), causing the rapid spread of symptoms without underlying pathophysiological cause [16, 17]. This demonstrates that social learning has the capacity to take hold in situations where there is potential for rapid spread (e.g., in online environments). However, the underlying mechanisms of the effect are not well understood. Expectancies and anxiety have been hypothesized as mediators (i.e., the underlying mechanisms through which social learning leads to transmission of symptoms), but few studies have tested these links [e.g.,^16^, ^18^, ^19^]. Modulation is suggested to occur via trait empathy, but conflicting results exist [e.g., 8–11, 14].

Importantly, research to date has largely neglected social learning in online settings. Throughout the COVID-19 pandemic, there has been a staggering increase in the frequency of social interactions taking place through online platforms [20]. For example, between the end of 2019 and April 2020, the use of the video-conferencing application Zoom increased by 2900% [2]. As these platforms are highly prevalent in education [21] and healthcare settings [22], there exists significant potential for the exacerbation of symptoms via online social transmission. Further, despite the relevance of social learning in the community-level transmission of symptoms, existing experimental research has been primarily focused on the singular model–observer interactions, which lack ecological validity. For example, in real-world situations, a person may inform their friend that they experienced food poisoning at a dinner they both attended. The friend may then go on to video call another friend who was present at that same dinner, reporting that they too are now feeling unwell. Moreover, in such studies, there is only a single model who is also typically an actor explicitly aware of the experimental aims (see Ref. [23] for an exception). It is, therefore, essential to investigate whether socially induced symptoms can be generated through online interactions, particularly in scenarios where social transmission stretches beyond a single social dyad.

The present study, therefore, examined the transmission of VR cybersickness online across a three-generation *social transmission chain* [24]. Cybersickness refers to the experience of symptoms such as nausea, stomach discomfort, and disorientation following immersion in VR [25]. The social transmission chain method provides a procedure for investigating group-level diffusion of information. The concept, frequently applied in cultural learning studies, explores the transfer of knowledge between people [26]. In lab-based settings, an experimenter provides a “First-Generation” participant with information. This participant is tasked with recalling this information for the “Second-Generation” participant. The Second-Generation does the same for the Third-Generation, and so on, thus establishing a social chain [27]. However, this method has never been applied to symptom transmission.

In the present study, the original “information” concerned adverse symptoms (i.e., cybersickness) experienced in reaction to a VR experience, modeled by an actor. Each participant in the chain sequentially underwent the same VR experience after viewing the generation prior. An exacerbation of cybersickness in First-Generation participants, relative to a control group without social observation, demonstrates the transmission of symptoms from an actor to a naïve participant in an online setting. Social transmission along the chain can then be tested by observing whether any adverse reactions experienced by First-Generation participants are propagated to the Second and Third-Generations. As information transmitted through social chains becomes increasingly distorted, inaccurate, and shorter as it progresses [28], it was hypothesized that the First-Generation group would experience the greatest increase in cybersickness, followed by the Second then Third-Generation. Anticipatory anxiety and expectancy were explored as mediators, and trait empathy as a moderator, of socially transmitted symptoms.

## Methods

Experimental design and analyses were pre-registered (see: https://aspredicted.org/me8j8.pdf). Ethical approval was granted by the University of Sydney Human Research Ethics Committee (Protocol 2020/198). Supplemental material provides a full diagram of recruitment procedures.

### Participants

In total, 177 healthy participants took part (mean age = 27 years, range = 18–61; *SD* = 6, 105 self-reported as female, 70 male, and 2 “other”). Information regarding sample SES and race/ethnicity was not collected. Australia-wide recruitment was conducted via Facebook and the University of Sydney CareersHub. Consistent with our prior research [29, 30], participants with >10 prior VR exposures, a medical condition increasing postural instability or risk of nausea, who were pregnant, had epilepsy, a pacemaker, or preexisting binocular visual abnormalities were not eligible to take part. Participants were required to have access to a smartphone with a diagonal screen size of 4.1–6.1 inches running the latest YouTube application. Informed consent was obtained from all individual participants included in the study. All participants were debriefed upon the conclusion of the study and provided with a VR headset (details below) which they kept as compensation for their time.

### Design

**Figure 1.**
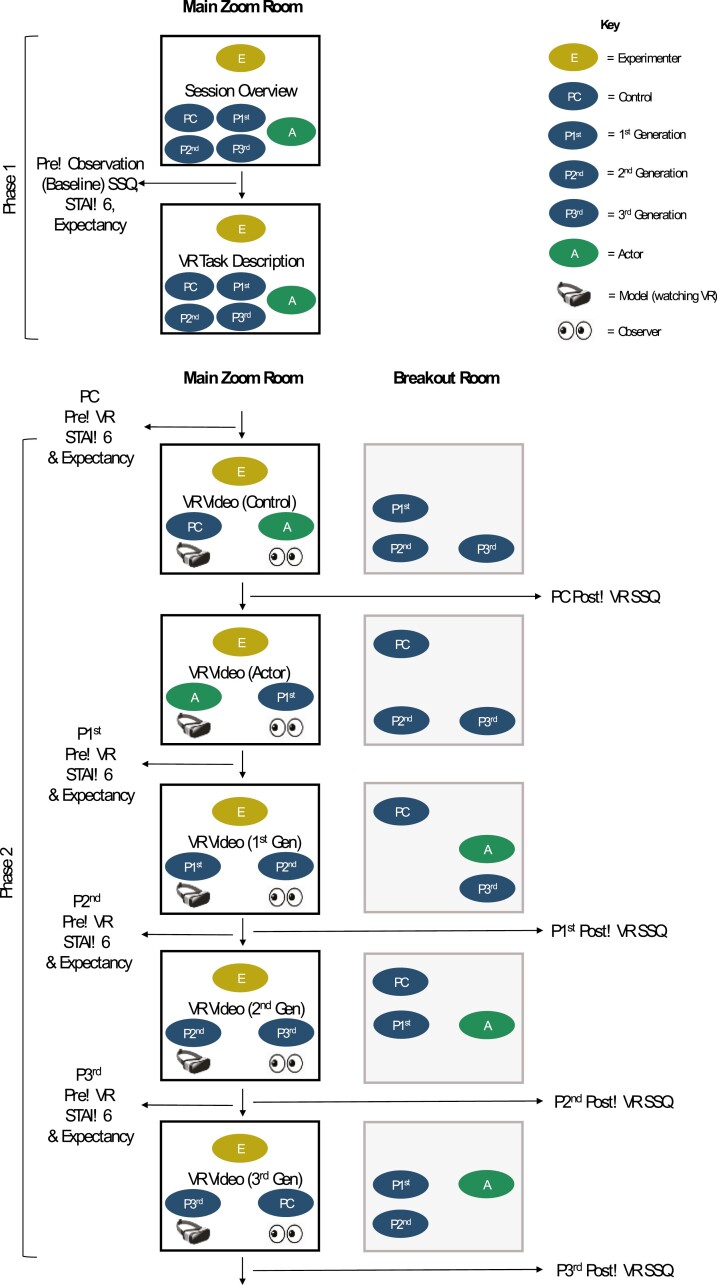
(LEGEND) *Flowchart of the Experimental Procedure. Note.* The social modeling procedure was complete at the end of Phase 2.

Participants were recruited under the guise that the study investigated online learning in VR. The true purpose was to examine virtually transmitted cybersickness. Testing took place via the video-conferencing application Zoom, with all participants watching the same VR roller coaster. A single-factor between-subjects design was employed (see Fig. 1), with participants randomly allocated (via random number generator) to four transmission groups: (1) First-Generation, (2) Second-Generation, (3) Third-Generation, or (4) Control (i.e., no social modeling). First-Generation participants experienced the VR roller coaster after observing an actor model cybersickness. The same was true for subsequent generations, except that the Second-Generation viewed the First-Generation, and Third-Generation the Second-Generation. The Control group was positioned first in the social modeling procedure and did not observe social modeling prior to their VR experience. The primary outcome was cybersickness. State measures of anticipatory anxiety and expectancy were measured as potential mediators, and trait empathy as a potential moderator, of virtually transmitted cybersickness.

### Materials and Measures

#### Virtual reality

Shinecon VR G034A head-mounted displays were employed. Participants watched two VR videos: (1) a roller coaster ride; (2) footage of landmarks in Paris. The roller coaster video (see: https://youtu.be/keCG91jR0pU) was implemented as a “cybersickness inducing” stimulus. The Paris video (see: https://youtu.be/W9e9fRPZIhI) served to reinforce the cover story.

#### Social modeling

A male actor, who presented as another participant, modeled symptoms for First-Generation participants, while a female experimenter led the sessions. The same two individuals conducted all sessions. During the actor’s modeling phase, they verbalized and gestured three distinct symptoms of cybersickness (nausea, general discomfort, and sweating). Each symptom was modeled once during the video and verbally reported once after, for a total of three unique symptoms transmitted twice each. For consistency across sessions, the actor followed a set script (see https://youtu.be/x6BeQAOODPA).

#### Cybersickness

 Cybersickness was measured using the Simulator Sickness Questionnaire (SSQ; [31]; Cronbach’s α = 0.93). Participants rated their symptoms such as “general discomfort” and “nausea” using an eleven-point scale: 0 (*not at all*) to 10 (*severely*). A baseline measure was taken at the beginning of each session, with the active measure being taken immediately after the participant's own VR experience.

#### Verbal symptom reports

The symptoms verbally reported by participants during their modeling phase were recorded by the primary experimenter at the time of testing. A second researcher checked and validated these symptoms by viewing the session video recordings. Participant symptom reports were clear and there was no disagreement in the independent recordings made by the two researchers. A total score was calculated by tallying the number of symptoms reported. Repeated symptoms were tallied individually (e.g., if dizziness was mentioned twice, it was tallied twice).

#### Anticipatory anxiety

 Anxiety was assessed using the short-form State-Trait Anxiety Inventory (STAI-6; [32]; Cronbach’s α = 0.82), which includes items such as “I am relaxed” and “I am upset”, rated on a four-point scale: 1 (*not at all*) to 4 (*very much*).

#### Expectancy for cybersickness

 Expectancy was assessed with the following item: “How much do you expect to experience feelings of cybersickness (e.g., nausea, general discomfort, sweating) during the VR video?” Ratings were on an 11-point scale: 0 (*not at all*) to 10 (*severely*).

#### Trait empathy

 The Interpersonal Reactivity Index (IRI; [33]; Cronbach’s α = 0.55) measured empathy. Participants rated how much they agreed with statements such as “Being in a tense emotional situation scares me” on a five-point scale: 0 (*does not describe me well*) to 4 (*describes me very well*).

### Procedure

#### Demographics and IRI

 Participants completed demographic information and the IRI via Qualtrics following consent. They subsequently signed up for a 1-h Zoom session and were posted a VR headset.

#### Baseline

The Zoom session started by informing participants of the study structure before they completed baseline assessments of the SSQ, STAI-6, and expectancy measures via Qualtrics. Once completed, participants were told that: (1) they would be watching two VR videos (the first in pairs, the second as a group); (2) that they would complete the paired task in a randomized order; (3) they would wait in a breakout room until it was their turn to participate. Participants were then randomized to one of the four experimental groups and sent to the breakout room.

#### Social transmission chain

Social modeling took place via live webcam interactions. In each phase of the chain, the experimenter and two participants were present in the main Zoom room, while the three remaining participants waited in the breakout room (“participants” will now include the actor unless otherwise stated). To begin the modeling procedure, the Control participant completed the active STAI-6 and expectancy measures in the main room. Once completed, the actor joined the room. During this Control-to-Actor phase, the Control participant was the model and the actor was the observer. The experimenter instructed the Control participant to verbalize their experience while watching the roller coaster video in their headset. Once the video ended, the experimenter asked the Control participant to describe how they felt physically during the video. The Control participant then returned to the breakout room and completed the active SSQ measure via Qualtrics. This provided an estimate of cybersickness induced by the VR stimulus. To reinforce the cover story, they also answered four dummy memory-based questions regarding the video.

The social transmission chain then began with the Actor-to-First-Generation phase, where the actor gestured and verbalized the experience of cybersickness symptoms while watching the video. Once the video ended, the experimenter asked the actor to describe how they felt physically during this video and the actor again described the experience of cybersickness symptoms. First-Generation participants then completed the active STAI-6 and expectancy measures while the actor returned to the breakout room. Upon finishing these measures, the First-Generation-to-Second-Generation phase began with the First-Generation participants watching the same VR video while being observed by Second-Generation participants. First-Generation participants were asked to verbalize their experience while watching the VR roller coaster, subsequently asked to describe how they felt physically in front of the Second-Generation participants and completed the active SSQ measure thereafter. This phased process continued with the Second-Generation being observed by the Third-Generation and concluded with the Third-Generation being observed by the Control group. All participants therefore both modeled and observed once each. In sessions with less than four participants, the Control participant observed the most distal generation (e.g., Second-Generation if one participant was missing).

Once the modeling procedure was complete, participants returned to the main room and watched the second video as a group. Participants were required to collaboratively answer three dummy memory-based questions regarding the video content. However, this part of the study was simply to uphold the cover story and served no purpose to the present data. Following this, participants completed a manipulation check via Qualtrics.

### Power and Data Analysis

Sample size was determined a priori. A minimum of 30 participants per group were required to achieve 80% power (α = 0.05) with an effect size of $\eta_{p}^{2}$ = 0.09, which is consistent with our previous research concerning cybersickness [29, 30] and the average number of participants included in existing social modeling research [e.g., 9–11, 14]. As the study required multiple participants per-session, a high attrition rate was expected. It was decided prior to commencing the study that sessions would proceed if at least two participants were present and that data collection would run until the 30 participant minimum was reached for the Third-Generation group.

Participants with extreme baseline cybersickness (>3SD above the mean, *n* = 4) were excluded based on pre-registered criteria. Five further participants were excluded due to technical difficulties, inability to follow instructions, or withdrawal. Primary analysis was a one-way ANCOVA with the between-subjects factor of Generation as the independent variable and active SSQ score as the dependent variable. Covariates were baseline SSQ score and dummy-coded gender. Gender was included as a covariate as previous studies have shown that gender-related factors moderate socially-acquired symptoms and pain [e.g., 8, 14]. Planned orthogonal contrasts were: (1) no social modeling (Control) versus social modeling (all Generations combined); (2) actor modeling (First-Generation) versus peer modeling (Second/Third-Generation combined); (3) Second versus Third-Generation modeling. It was hypothesized that the strength of socially acquired symptoms would weaken throughout the chain. It has been repeatedly demonstrated that a proportion of individuals are non-responsive to manipulations exploring psychogenic outcomes [e.g., 34, 35]. Further, no prior evidence existed regarding the frequency of symptoms likely to be modeled at the Second and Third-Generations of the chain, meaning that the overall strength of transmission could not be estimated. As such, an exploratory analysis was pre-registered whereby the symptoms verbalized by each model to subsequent participants (i.e., the verbal symptom reports described above) would be employed to determine how the frequency of side effects modeled by the actor changed across the generations (more information is included in the *post hoc* cybersickness analyses section below).

ANCOVAs, with the same planned orthogonal contrasts as the primary analysis, were performed on the dependent variables of symptoms modeled/verbally reported by participants, anticipatory anxiety, and expectancy measures. Anticipatory anxiety and expectancy were additionally investigated as potential mediators of social modeling on cybersickness. Trait empathy was investigated as a potential moderator using hierarchical linear regression where active SSQ score was the outcome, baseline SSQ and gender (dummy coded into two variables with male as the reference group: male vs. female, male vs other) were entered in the first step, orthogonal contrasts (First vs. Second/Third-Generation combined and Second vs. Third-Generation) and IRI score were entered in the second step, and the interaction between contrasts and IRI score entered in the third step. The control group was removed from this analysis as they did not observe a model and thus empathy should not play a role in their experience. Mediation analysis was conducted using Model 4 of SPSS PROCESS macro v3.5 [36]. In all mediation analyses, bootstrapping with 10,000 samples was conducted to determine 95% confidence intervals (CIs). These CIs determined statistical significance. All other statistical analyses were performed using SPSS Version 24 with α = 0.05.

## Results

### Baseline Group Differences

As shown in Table 1, there were no significant between-group differences in age, IRI, baseline measures (SSQ, STAI-6, and expectancy), gender, or prior VR experience (all *ps* > 0.05).

**Table 1:** Between-group differences in baseline characteristics

Baseline Measures											
	Control		First Generation		Second Generation		Third Generation				
	M	SD	M	SD	M	SD	M	SD	*F* (3,164)	p	
Age	26.36	5.34	26.02	5.38	27.68	7.34	26.55	5.09	0.64	0.59	
SSQ	8.21	9.87	9.76	9.10	6.65	7.72	8.23	8.53	0.91	0.44	
STAI-6	9.68	2.35	9.94	2.79	9.23	3.10	10.23	2.80	0.88	0.45	
Expectancy	2.62	2.72	2.68	2.06	2.48	2.64	3.10	2.37	0.40	0.75	
IRI	70.40	12.35	70.06	12.42	66.53	11.93	71.26	10.45	1.18	0.32	
**Demographic Information**											
	*n*	%	*n*	%	*n*	%	*n*	%	Fisher’s exact test	*p*	*Cramer’s V*
Gender									3.56	0.78	0.13
Male	19	40.4	17	34	17	42.5	13	41.9			
Female	28	59.6	31	62	23	57.5	18	58.1			
Other	0	0	2	4	0	0	0	0			
Prior VR Experience (hours)									3.80	0.72	0.11
0 hours	20	42.6	24	48	18	45	14	45.1			
1–5 hours	26	55.3	21	42	20	50	16	51.7			
6–10 hours	1	2.1	5	10	2	5	1	3.2			

### Cybersickness

**Figure 2.**
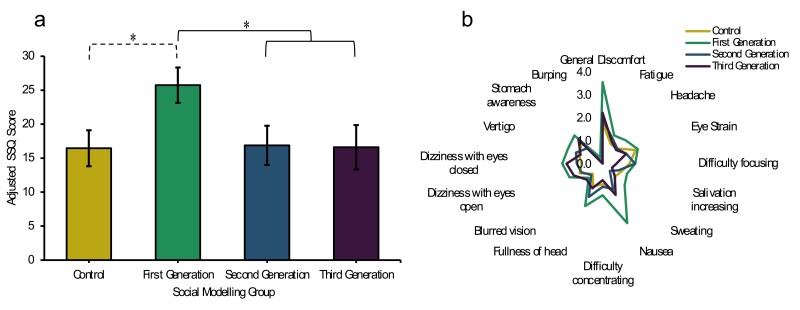
(LEGEND).*Active Cybersickness and Simulator Sickness Questionnaire Item Ratings of each Social Modeling Group. Note.* (a) Bars represent adjusted mean active SSQ scores (±1 SEM). Means adjusted for baseline SSQ = 8.30, gender (male versus female) = 0.59, and gender (male vs. other) = 0.12. Significant planned (full) and post hoc (dashed) contrasts are indicated by brackets. (b) Points represent adjusted mean active SSQ ratings for individual items between social generation groups. Individual item means adjusted for baseline SSQ and gender.

As shown in Fig. 2A, a significant main effect of group was observed on cybersickness ratings, *F*(3,161) = 2.84, *p* = 0.040, $\eta_{p}^{2}$ = 0.05. Orthogonal contrasts revealed significantly higher SSQ scores among the First-Generation (_adj_*M* = 25.74) relative to Second/Third-Generations combined (_adj_*M* = 16.75), *F*(1,161) = 6.92, *p* = 0.009, $\eta_{p}^{2}$ = 0.04. All other contrasts failed to reach statistical significance, all *ps* > 0.05.

The relationship between model symptom reports and observer cybersickness was also examined. First, participants’ verbal symptom reports were confirmed to be consistent with their self-report SSQ score (see Supplementary Material). As shown in Fig. 2B, there was a significant main effect of group on symptoms verbally reported by modeling participants to observing participants, *F*(3,161) = 4.970, *p* = 0.003, $\eta_{p}^{2}$  = 0.09. Contrasts revealed that the First-Generation group verbally reported more symptoms overall (_adj_*M* = 2.65) compared to Second/Third-Generations (_adj_*M* = 1.56, *F*(1,161) = 12.51, *p* = 0.001, $\eta_{p}^{2}$ = 0.07) and Control (_adj_*M* = 1.58, *F*(1,161) = 10.07, *p* = 0.002, $\eta_{p}^{2}$ = 0.06). The Second and Third-Generation groups did not significantly differ, *F*(1,161) = 0.374, *p* = 0.542, $\eta_{p}^{2}$ = 0.00.

### *Post hoc* Cybersickness Analyses

Unexpectedly, in the planned analysis, the contrast comparing cybersickness for those with any form of social observation (i.e., all generations) to Control was not significant, suggesting a lack of overall social transmission of cybersickness across the entirety of the chain. However, given that the First-Generation reported significantly greater cybersickness than subsequent generations, the lack of significance for the first planned contrast may have resulted from pooling across three generations. Two *post hoc* contrasts were therefore conducted. The first revealed that, relative to Control (_adj_*M* = 16.46), First-Generation participants (_adj_*M* = 25.74) reported significantly greater cybersickness, *F*(1,161) = 6.19, *p* = 0.014, $\eta_{p}^{2}$ = 0.04. The second revealed no difference in cybersickness between the Second/Third-Generations and Control *F*(1,161) = 0.01, *p* = 0.934, $\eta_{p}^{2}$ = 0.00.

The lack of a significant social modeling effect in the Second-Generation means that many in the Third-Generation would have observed only a limited number of symptoms being modeled. Similarly, while there was an effect of social modeling in the First-Generation, variance in the outcome means that some were more susceptible to social modeling than others. Consequently, those non-receptive would have transmitted fewer symptoms to the Second-Generation. In summary, social modeling would have weakened through the social-chain and no social transmission would be expected among participants who did not witness modeled symptoms. *Post hoc* analyses were therefore conducted to test whether social transmission occurred in Second and Third-Generation participants who saw symptoms modeled compared to those who did not.

Data from Second and Third-Generation groups were combined and separated into two strata dependent on the verbal symptom reports of the model they observed: (1) No Symptoms (*n* = 11); (2) Symptoms (*n* = 60). Independent samples *t*-tests were conducted comparing: (1) No Symptoms versus Symptoms [transmission effect]; (2) Control versus Symptoms [peer social modeling effect]; (3) Control versus No Symptoms [protective effect]. To control for covariates, the outcome (active SSQ) was regressed on Baseline SSQ and Gender and unstandardized residuals saved as the outcome (comparable to the original ANCOVAs). Within the Second and Third-Generations, SSQ scores differed between the No Symptoms versus Symptoms group (_adj_*M*_*D*_ = −11.38; *t*(69) = −2.272, *p* = 0.026), suggesting that participant-to-participant transmission occurred when the initial participant was receptive to social modeling. However, those in the Symptoms group did not differ from Control (_adj_*M*_*D*_ = 2.42; *t*(105)= − 0.751, *p* = 0.454), potentially due to the low number of verbal reports observed across the group (Median Observed Symptoms = 2, range = 1–8). Indicative of a protective effect of neutral modeling, those in the No Symptoms group reported lower cybersickness than the Control group (_adj_*M*_*D*_ = −9.93; *t*(56) = 2.018, *p* = 0.048). To quantify the evidence for or against an effect here, we calculated a Bayes Factor (BF) for this comparison using JASP V0.16 and the default Cauchy prior of width of *r* = 0.707. This indicated that it was three times more likely that those who saw a model report no symptoms experienced less cybersickness than Control (BF = 3.0).

### Anticipatory Anxiety

**Figure 3.**
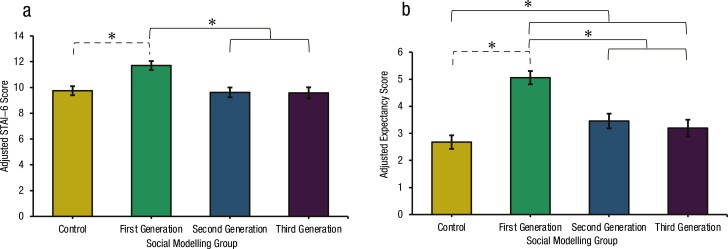
*Active Anticipatory Anxiety and Expectancy of each Social Modeling Group. Note.* (a) Bars represent adjusted mean STAI-6 scores (±1 SEM). Means adjusted for baseline STAI-6 = 9.75, gender (male vs. female) = 0.59 and gender (male vs. other) = 0.12. Significant planned (full) and post hoc (dashed) contrasts are indicated by brackets. (b) Bars represent adjusted mean expectancy scores (±1 SEM). Means adjusted for baseline expectancy = 2.69, gender (male vs. female) = 0.59 and gender (male vs. other) = 0.12. Significant planned (full) and post hoc (dashed) contrasts are indicated by brackets.

As shown in Fig. 3A, there was a significant main effect of group on active STAI-6 scores, *F*(3,161) = 8.19, *p* < 0.001, $\eta_{p}^{2}$ = 0.13. Contrasts demonstrated that the First-Generation group (_adj_*M* = 11.70) reported significantly higher STAI-6 scores relative to the Second/Third-Generations (_adj_*M* = 9.60), *F*(1,161)=21.63, *p* < 0.001, $\eta_{p}^{2}$ = 0.12, with greater anticipatory anxiety among the former. No other planned contrast reached statistical significance, all *ps* > 0.05. A single post hoc contrast revealed that the First-Generation group (_adj_*M* = 11.70) reported significantly higher STAI-6 scores compared to Control (_adj_*M* = 9.75), *F*(1,161) = 15.63, *p* < 0.001, $\eta_{p}^{2}$ = 0.09.

### Expectancy

As shown in Fig. 3B, expectancy for cybersickness also differed between groups, *F*(3,161) = 16.74, *p* < 0.001, $\eta_{p}^{2}$ = 0.24. Planned contrasts demonstrated that groups who witnessed social modeling (_adj_*M* = 3.90) reported significantly higher expectancies relative to Control (_adj_*M* = 2.68), *F*(1,161) = 16.75, *p* < 0.001, $\eta_{p}^{2}$ = 0.09. The First-Generation group (_adj_*M* = 5.06) also reported significantly higher expectancies compared to the Second/Third-Generation groups (_adj_*M* = 3.23), *F*(1,161) = 29.03, *p* < 0.001, $\eta_{p}^{2}$ = 0.15. The Second- and Third-Generation groups did not significantly differ, *F*(1,161) = 0.405, *p* = 0.525, $\eta_{p}^{2}$ = 0.00. A single post hoc contrast revealed increased expectancies in the First-Generation relative to Control, *F*(1,161) = 45.84, p < 0.001, $\eta_{p}^{2}$ = 0.22.

### Mediation: Anxiety and Expectancy

**Figure 4.**
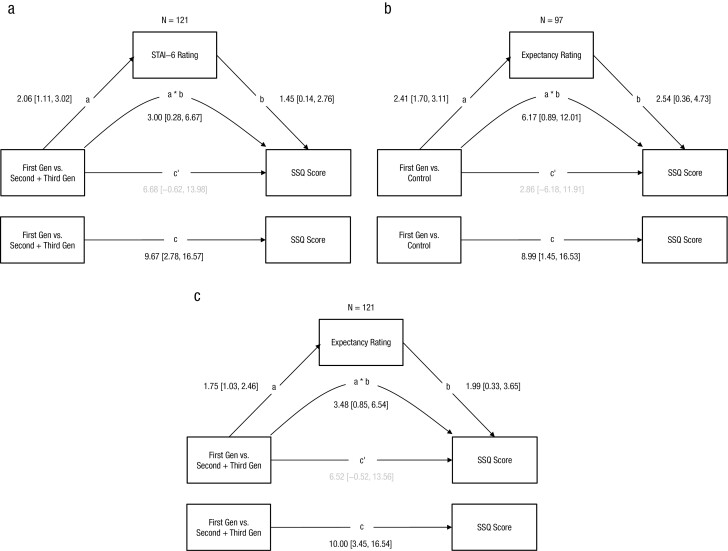
*Mediatory Effects of Anticipatory Anxiety and Expectancy of Cybersickness Note*. Values are unstandardized beta coefficients with 95% confidence intervals (CI). Figures represent mediatory effects of anticipatory anxiety (a) and expectancy (b and c). Covariates (baseline SSQ and gender) were included in the models but are not represented here for brevity. Paths *a*, *b*, and *c* are the direct paths between variables. Curved path a*b is the indirect effect with bootstrapped CIs. Path *cʹ* is the direct association between the group contrast and cybersickness, controlling for the indirect effect.

STAI-6 scores mediated the effect of group (First vs. Second/Third-Generations) on SSQ score (Fig. 4A), direct effect = 6.68 [95%CI = −0.62 to 13.98], and indirect effect = 3.00 [95%BootCI = 0.28 to 6.67]. This was not the case when comparing the First-Generation to Control: direct effect = 7.25 [95%CI = −1.35 to 15.85], and indirect effect = 1.71 [95%BootCI = −1.47 to 6.25].

Expectancy mediated the effect of First versus Second/Third-Generation Group on SSQ scores (Fig. 4B), direct effect = 6.52 [95%CI=−0.52 to 13.56] and indirect effect = 3.48 [95%BootCI = 0.85 to 6.54], as well as the effect of First-Generation versus Control Group on SSQ scores (Fig. 4C): direct effect = 2.86 [95%CI = −6.18 to 11.91] and indirect effect = 6.13 [95%BootCI = 0.89 to 12.02].

### Empathy

A hierarchical linear regression was performed to determine whether trait empathy moderated the effect of social modeling on cybersickness. Neither IRI scores, nor their interaction with the social modeling group, predicted SSQ scores (all *ps* > 0.05), potentially due to the low internal consistency of the scale in the present study (see “Methods” section). The full regression model is included as supplemental materials.

## Discussion

The present study investigated the social transmission of cybersickness in an online setting, with three novel findings: (1) exacerbated cybersickness among First-Generation participants demonstrates that reported symptoms we witness online can modulate our own health outcomes; (2) symptom experiences were mediated by negative expectancies and state anxiety, indicating that these are key underlying mechanisms driving the transmission of symptoms in online settings; and (3) virtual transmission among Second and Third-Generation participants was dependent on what the previous participant reported, where witnessing neutral modeling (i.e., no symptoms reported) may have a protective effect.

In what was the first exploration of symptom propagation through a social chain, transmission was observed in the First-Generation group. This is consistent with existing evidence that socially acquired information can shape expectations, leading to a worsening of health outcomes [16, 18, 19]. However, to our knowledge, this is the first study to show that symptoms can be induced when observation takes place from an online interaction (i.e., Zoom). These findings have important implications given the increasing use of online platforms to disseminate health-related information [37], where witnessing negative outcomes may worsen observer prognosis. Interestingly, there was no overall social transmission of symptoms in Second and Third-Generations further along the chain. Instead, only first-hand observation of the initial model/actor was sufficient to elicit symptom transmission. However, the actor transmitted a uniform and high frequency of symptoms (*N* = 6). Exploratory pre-registered analyses within Second and Third-Generation groups indicated that those who witnessed a peer-participant report any symptoms experienced greater cybersickness than those who did not. However, those that witnessed peer-modeled symptoms did not differ from the Control group. Given the average number of symptoms witnessed in the Second and Third-Generations was relatively low, this may have attenuated socially transmitted cybersickness. It also appeared that the “no symptoms observed” group experienced less cybersickness than even the Control group. It is thus possible that the neutrally valenced modeling served as a protective factor, whereby participants may have altered their perceptions regarding the cybersickness-inducing qualities of the VR stimulus to be less than originally thought. Symptom experience, therefore, appeared related to the initial participant’s receptivity to the social modeling effect. Interventions that directly lower negative expectancies among receptive participants may therefore lead to cessation of the chain.

While extrapolation of results should be treated with caution due to the exploratory nature of the analyses, we propose that the social transmission of symptoms will be most virulent in situations where strong negative expectancies exist across individuals and that non-receptive individuals could act as circuit-breakers. This is particularly pertinent in health-related settings, such as the current administration of COVID-19 vaccines, where expectancies for side effects are well defined and directly relevant to the observer. Here, what we communicate to each other online may directly impact our physical experience. We recommend that future research should therefore focus on such scenarios.

Anticipatory anxiety, expectancy, and trait empathy were explored as individual characteristics influencing socially induced symptoms. Two studies have previously demonstrated that expectancy mediates the experience of socially modulated pain [18, 19]. However, those studies involved a concomitant social modeling and conditioning procedure, rather than exploring social modeling in isolation. Two further studies found no correlation between anxiety [9] or expectancy [11], and socially induced hyperalgesia. However, methodological limitations in these studies, such as measuring anxiety prior to social modeling, and expectancy retrospectively, made it difficult to gauge their effects. In this study, we found that those who observed the actor’s modeling were more anxious and had higher negative expectancies than those who observed peer modeling (i.e., another participant), who on average reported fewer symptoms. Moreover, expectancy also mediated symptoms in the First-Generation relative to Control. This mediatory effect found with expectancy but not anxiety could be attributed to genuine differences in their psychological influence. However, while the expectancy measure was explicitly related to the participant’s VR experience, the anxiety measure reflected the participants’ general psychological state. As such, the present outcomes may not represent anticipatory anxiety about experiencing cybersickness from VR per-se. The use of a specific anxiety measure may therefore produce more rigorous findings in the future.

Trait empathy was found not to moderate cybersickness. Prior research has shown associations between empathy and socially acquired symptoms following in-person social modeling [8, 14], but not via prerecorded video [11, 38]. Consequently, it is possible that the Zoom interface degraded the perceptual information necessary to facilitate any association between empathy and social transmission. A direct comparison of in-person and video-based modeling using alternative measures of empathy is necessary to clarify these results. It is also possible that this finding was due to the low internal inconsistency of the IRI in the current study. Nevertheless, in light of this, an understanding of *who* is likely to be at risk from virtually transmitted symptoms is still crucial. Expectancy and anxiety underpinned the experience of cybersickness, but this does not address the underlying reason why some developed these state-related characteristics to a greater extent than others. Future research should strive to understand the dispositional factors that drive these differences to identify individuals at risk of adverse health outcomes following social observation.

There are a number of strengths to the present study. Here we show that an experimental VR model [see 29, 30] is capable of facilitating socially induced cybersickness. In addition, we demonstrate the viability of software such as Zoom for experimentation and provide preliminary evidence to suggest that symptom experiences could be virtually transmitted by genuine participants who are unaware of the experimental outcomes. This finding is reinforced by the wide-ranging community sample of varying ages. However, limitations of the present study should be noted. Temporal disparity between baseline and active measures collected between the groups may have introduced noise to the cybersickness, anxiety, and expectancy measures. Moreover, given the nonspecific nature of the STAI-6 scale, the active measure for the Control group may have also included any additional anxiety experienced resulting from being the first in the procedure. Further, the existing literature has shown that female viewers are more susceptible to these socially modeled effects, particularly when the model themselves are female (see Ref. 39] for a recent review). In this study, only a male model was used, thus making it difficult to ascertain the influence of gender.

In summary, the current study demonstrates that symptoms modeled via online platforms can be transmitted to the observer. While further propagation of symptoms along a social transmission chain remains ambiguous, witnessing a model who was not receptive seemed to serve as a protective factor that could be harnessed to block social transmission. Given that these latter outcomes are exploratory, further research is needed. Negative expectancies and anxiety were identified as significant mediators, shedding light on the underlying mechanisms facilitating socially transmitted symptoms, with important implications for their future reduction via these routes. These outcomes have significant implications for how health information is circulated across social circles and demonstrate the need for users to be aware of how they apply information accessed online (e.g., from live video calls with friends and family, to Zoom meetings, or even live-streams) to their own health experiences and be prudent in how they disseminate this information to others. Future research may seek to gain a greater understanding of the dispositional factors related to the social model and how it relates to the transmission of symptoms. Further exploration of how the relationship between the model and observer influences these socially induced effects would also be valuable. For example, whether an observer applies the socially learned information to themselves may depend on the degree to which they trust the model and/or believe the model is a credible source of information.

## Supplementary Material

kaac081_suppl_Supplementary_Figure_S1

kaac081_suppl_Supplementary_Tables_S1

kaac081_suppl_Supplementary_Material
