# Supplementary figures and images for: Peer-to-peer: The Social Transmission of Symptoms Online
Source: Ann Behav Med. 2023 Apr 10;57(7):551–60. doi: 10.1093/abm/kaac081 (PMC10312298; doi:10.1093/abm/kaac081)

1 **Supplemental Material Figure 1**  
 2 *CONSORT Flow Diagram of the Participant Recruitment Process*

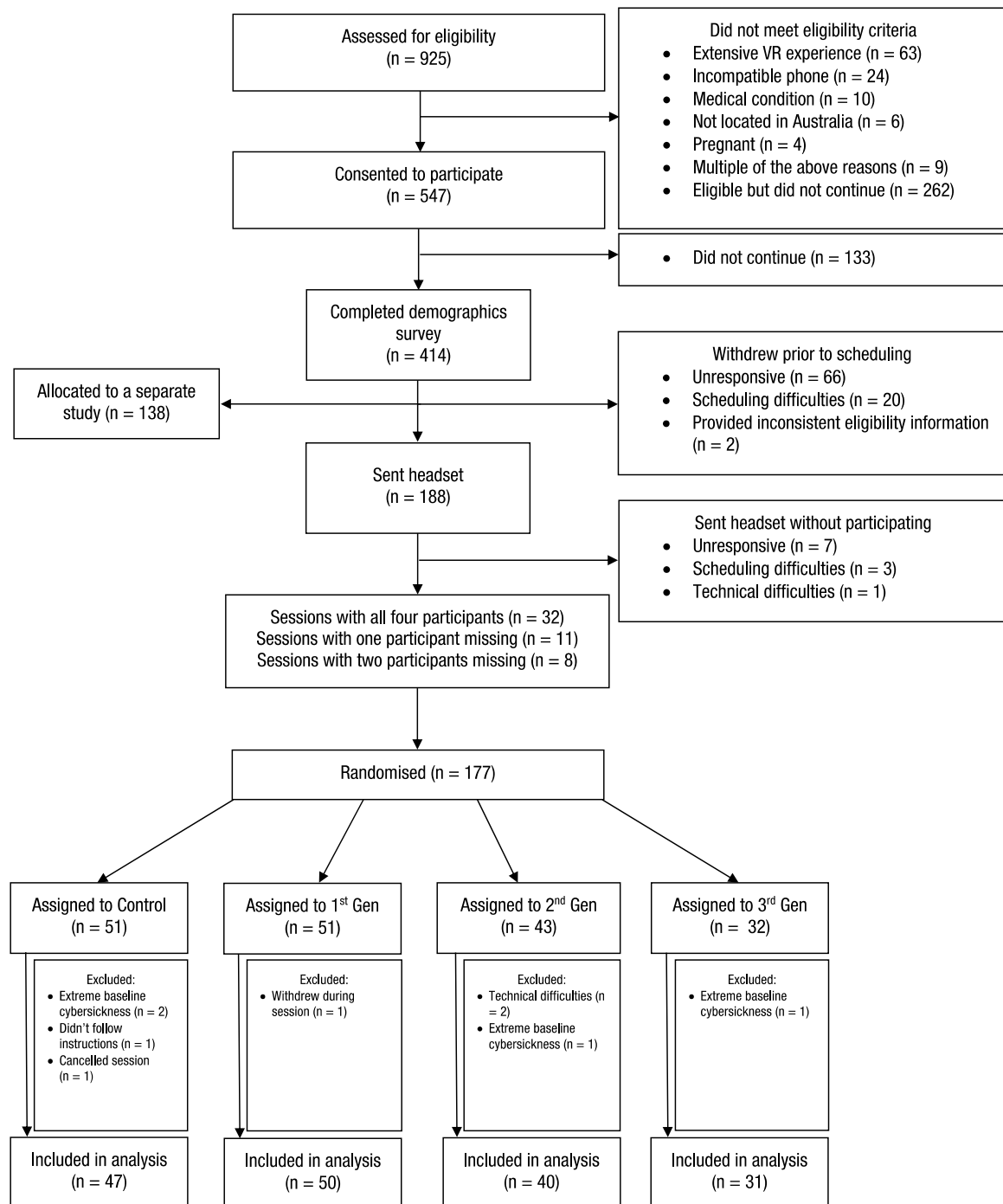

Supplement: kaac081_suppl_Supplementary_Figure_S1 [file kaac081_suppl_supplementary_figure_s1.pdf]
