# Supplementary material for: Peer-to-peer: The Social Transmission of Symptoms Online
Source: Ann Behav Med. 2023 Apr 10;57(7):551–60. doi: 10.1093/abm/kaac081 (PMC10312298; doi:10.1093/abm/kaac081)
Supplement: kaac081_suppl_Supplementary_Tables_S1 [file kaac081_suppl_supplementary_tables_s1.pdf]

Supplemental Material Table 1

Empathy Hierarchical Regression Model

Variable	*B*	*SE B*	β	*ΔR²*
**Step 1**				.058
Baseline SSQ	.432	.205	.192*	
Gender 1	4.524	3.586	.116	
Gender 2	5.127	13.737	.034	
**Step 2**				.070*
Baseline SSQ	.357	.202	.159	
Gender 1	5.948	3.869	.153	
Gender 2	2.765	13.839	.018	
Group (First vs. Second/Third Gen)	-9.708	3.487	-.249**	
Group (Second vs. Third-Gen)	-1.099	4.483	-.022	
IRI	-1.86	.160	-.114	
**Step 3**				0.022
Baseline SSQ	.322	.202	.143	
Gender 1	6.251	3.862	.160	
Gender 2	3.696	13.885	.025	
Group (First vs. Second/Third Gen)	-8.999	3.501	-.231**	
Group (Second vs. Third-Gen)	-1.193	4.514	-.024	
IRI	-.238	.168	-.147	
Group (First vs. Second/Third Gen) x IRI	-.001	.295	.000	
Group (Second vs. Third Gen) x IRI	.676	.407	.152	

**p<.05, **p<.01*
