# Supplementary material for: Peer-to-peer: The Social Transmission of Symptoms Online
Source: Ann Behav Med. 2023 Apr 10;57(7):551–60. doi: 10.1093/abm/kaac081 (PMC10312298; doi:10.1093/abm/kaac081)
Supplement: kaac081_suppl_Supplementary_Material [file kaac081_suppl_supplementary_material.pdf]

Supplemental Material Model 1

Participant Verbal Reports and SSQ Score

Controlling for baseline SSQ and gender, the overall regression between participant verbal symptom reports and their own SSQ score was significant, R^2=.349, $F(4,163)$=21.81, p<.001. In this model, the symptoms reported by the participant was a significant predictor of their reported SSQ score (β=.482, p<.001), suggesting that the symptoms they verbalised in front of the observer was an accurate reflection of their own reported cybersickness.
